# Seaweeds: an opportunity for wealth and sustainable livelihood for coastal communities

**DOI:** 10.1007/s10811-014-0304-8

**Published:** 2014-05-03

**Authors:** Céline Rebours, Eliane Marinho-Soriano, José A. Zertuche-González, Leila Hayashi, Julio A. Vásquez, Paul Kradolfer, Gonzalo Soriano, Raul Ugarte, Maria Helena Abreu, Ingrid Bay-Larsen, Grete Hovelsrud, Rolf Rødven, Daniel Robledo

**Affiliations:** 1 Bioforsk, Norwegian Institute for Agricultural and Environmental Research, Frederik A. Dahlsvei 20, 1430 Ås, Norway; 2 Department of Oceanography and Limnology, Federal University of Rio Grande do Norte, Via Costeira, Praia de Mãe Luiza, s/n, Natal, RN 59014-002 Brazil; 3 Instituto de Investigaciones Oceanológicas, Universidad Autónoma de Baja California, Km 107 Carretera Tijuana-Ensenada, 22860 Ensenada, BC Mexico; 4 Aquaculture Department, Federal University of Santa Catarina, Rod. Admar Gonzaga 1346, Itacorubi, Florianópolis, SC Brazil; 5 Facultad de Ciencias del Mar, Centro de Estudios Avanzados en Zonas Aridas, Universidad Católica del Norte, Coquimbo, Chile; 6 PSW SA, Av. Paul Poblet Parcela D19 Lote 1, Lurín, Lima 16, Peru; 7 Soriano SA, JC Evans 40, 9105 Gaiman, Chubut Argentina; 8 Acadian Seaplants Limited, 30 Brown Ave., Dartmouth, NS B3B-1X8 Canada; 9 ALGAPlus Lda, Travessa Alexandre da Conceição 3830-196, Ílhavo, Portugal; 10 Nordland Research Institute, Bodø, Norway; 11 Department of Marine Resources, CINVESTAV-IPN, Km 6 Carretera Ant. Progreso, 97310 Mérida, Yucatán Mexico; 12 Present Address: Arctic Agriculture and Land Use Division, Bioforsk, NO-8049 Bodø, Norway

**Keywords:** Coastal management, Latin America, Marine resources, Seaweed industry

## Abstract

The European, Canadian, and Latin American seaweed industries rely on the sustainable harvesting of natural resources. As several countries wish to increase their activity, the harvest should be managed according to integrated and participatory governance regimes to ensure production within a long-term perspective. Development of regulations and directives enabling the sustainable exploitation of natural resources must therefore be brought to the national and international political agenda in order to ensure environmental, social, and economic values in the coastal areas around the world. In Europe, Portugal requires an appraisal of seaweed management plans while Norway and Canada have developed and implemented coastal management plans including well-established and sustainable exploitation of their natural seaweed resources. Whereas, in Latin America, different scenarios of seaweed exploitation can be observed; each country is however in need of long-term and ecosystem-based management plans to ensure that exploitation is sustainable. These plans are required particularly in Peru and Brazil, while Chile has succeeded in establishing a sustainable seaweed-harvesting plan for most of the economically important seaweeds. Furthermore, in both Europe and Latin America, seaweed aquaculture is at its infancy and development will have to overcome numerous challenges at different levels (i.e., technology, biology, policy). Thus, there is a need for regulations and establishment of “best practices” for seaweed harvesting, management, and cultivation. Trained human resources will also be required to provide information and education to the communities involved, to enable seaweed utilization to become a profitable business and provide better income opportunities to coastal communities.

## Introduction

The worldwide seaweed industry provides a wide variety of products for direct or indirect human uses that have an estimated total value of US$10 billion per year (Bixler and Porse 2011; FAO 2013). Sea vegetables for human consumption constitute about 83 % of production (Craigie 2011), while the remainder is used as fertilizers and animal feed additives, medical applications (Zimmermann et al. 2005; Ehrhart et al. 2013), and biotechnological applications (McHugh 2003). Worldwide, macroalgal production increases 5.7 % every year and more than 18 million tons of macroalgae were produced from global capture and aquaculture in 2011 (FAO 2014). In 2011, 96 % of the global total production of macroalgae came from aquaculture, with Asian countries dominating seaweed culture production (99.05 % by quantity and 99.36 % by value, FAO 2014). Five genera (e.g., *Saccharina*, *Undaria*, *Porphyra*, *Eucheuma*/*Kappaphycus*, and *Gracilaria*) represented around 98 % of the world’s cultivated seaweed production (Suo and Wang 1992; Pereira and Yarish 2008). *Saccharina japonica* was the most cultivated algae in the world until 2010 when the production of *Eucheuma*/*Kappaphycus* reached over 5.5 million tons for a value over US$1.3 billion (Suo and Wang 1992; McHugh 2003; FAO 2014). *Saccharina* and *Eucheuma*/*Kappaphycus* are mostly produced as raw materials for the food and food polymer industries. Aquaculture of seaweed is scarce outside of Asia, which triggered a worldwide search for hitherto unexploited natural seaweed resources. In 2011, 786,466 t of seaweeds was commercially harvested in 28 countries, ranging from cold to tropical coastlines in both hemispheres, with over 55 % of the biomass harvested in Latin America and almost 32 % in Europe (FAO 2014). The top producers are Chile and Norway respectively accounting for 51.3 and 19.2 % of the global catches of natural seaweed (FAO 2014).

The Pacific coast of South America is naturally rich in marine resources (Thiel et al. 2007; Ortiz 2008; Taylor et al. 2008; Vásquez et al. 2012; Vega et al. 2013). In Latin America, macroalgae are an important group for species richness of all regions, varying from 4.9 to 8.7 % of total marine species biodiversity (O’Dor et al. 2010). The highest biodiversity of macroalgal species is reported for the Brazilian region (10.6 species per 100 km of coast), followed by the Humboldt Current system (7.3 species per 100 km of coast), the Tropical West Atlantic (7.1 species per 100 km of coast), and the Tropical East Pacific (6.0 species per 100 km of coast), with the lowest diversity attributed to the Patagonian Shelf (4.7 species per 100 km of coast; Miloslavich et al. 2011). The trends reported so far, however, may not truly reflect real patterns, as sampling has not been equal throughout the continent, and taxonomic capacity is very uneven from one country to another, as is the case in the Caribbean (Robledo and Townsend 2006; Miloslavich et al. 2010). In this context, there are still some efforts to be made on basic research to describe and evaluate some of the macroalgal resources in the region (Critchley et al. 2006).

Macroalgae or seaweeds are one of major components of primary biomass production in coastal maritime ecosystems and play an essential ecological role as habitat and substrata for invertebrates, fish, mammals, and birds (Vásquez 1992; Graham et al. 2007). Drastic reduction of any macroalgal community directly influences marine biodiversity, as well as reproduction, recruitment, and growth rates of marine fauna (Vásquez and Santelices 1984; Vásquez 1993). Furthermore, macroalgae may also protect coastlines against erosion (Dayton et al. 1984) and contribute significantly to the marine carbon cycle (Thiel et al. 2007; Ugarte et al. 2010; Vásquez et al. 2013). It is possible that overexploitation of natural seaweed resources could lead to significant ecological, economic, and social consequences at local, regional, and even global scales (Graham et al. 2007; Rebours and Karlsen 2007). In this regard, Alvarez and Vodden (2009) examined the relationships between human actors and *Chondracanthus chamissoi* harvesting in the community of Pisco in Peru. By using local ecological knowledge (LEK), these authors found that a disorderly extraction of *C. chamissoi* generates reductions in the resource that could be exacerbated by climatic change (“El Niño” and “La Niña” phenomena) and the rupture of ecological cycles due to pollution of the marine space and decline of marine species.

Accurate data about seaweed aquaculture or the exploitation of the natural resource stock are difficult to obtain through the international channels in most of the Latin American countries (except in Chile). The necessary information was extracted from database queries at the Harvest data from Instituto de Fomento Pesquero (IFOP) in Chile; Secretaría de Agricultura, Ganadería, Desarrollo Rural, Pesca y Alimentación (SAGARPA) in Mexico; numerous Dirección Regional de Produce (DIREPRO) in Peru; and the FAO Fisheries and Aquaculture Information and Statistics Service to discuss the trends for some Latin American countries, especially for those harvesting seaweeds. The potential for seaweed aquaculture, particularly for red seaweeds, has been addressed in Hayashi et al. (2013). In Latin America, exploitation of seaweed happened or has happened in Argentina, Brazil, Chile, Mexico, Peru, Uruguay, and Venezuela (Critchley et al. 2006; FAO 2013). The main seaweed exploitation has been reported in Argentina, Brazil, Chile, Mexico, and Peru. In these countries, traditional harvesting and/or aquaculture is presently active or has been attempted and has generated an estimated biomass volume around 554,585 t in 2009 (equivalent to US$115 million in value), with an estimated decline in total volume to 444,686 t in 2011 (equivalent to US$22 million; Table 1). The main objective of this study is to present a short overview of the management and regulations in place for the exploitation and harvesting of seaweeds in the five above-mentioned Latin American countries. Some of the environmental, social, economic, and political obstacles to sustainable development for seaweed utilization in Latin America will be identified through multidisciplinary analyses, combining natural, ecological, and social sciences. The European Environmental Agency (EEA) also stressed the need for an analytical approach for coastal areas and to place this in an ecosystem-based management approach combining integrated spatial planning and management. Included in this is the need for a consolidated knowledge base and widespread information sharing to support informed policy development and management actions (EEA 2013). Examples for long-term management from Europe (Norway, Portugal) and North America (Canada) are then shortly presented, and successful practices in these Northern regions are then identified and presented as possible as “best practices” model for long-term sustainable seaweed exploitation in Latin America. Particular emphasis is given to identify the possible actions in particular to overcome social, economic, and political obstacles for using Latin American seaweeds as an economic resource for wealth and sustainable livelihoods for coastal communities.

**Table 1:** Harvesting of seaweeds in Argentina, Brazil, Chile, Mexico, and Peru and commercial aquaculture of seaweeds attempted in only four of these countries. Harvest and aquaculture volumes of seaweed biomass in tons

	2000	2001	2002	2003	2004	2005	2006	2007	2008	2009	2010	2011
Seaweed harvest^a^												
Argentina	3											
Brazil												
Chile	280,844	299,759	315,660	349,128	410,850	425,343	334,674	337,206	406,223	456,184	380,742	417,965
México	33,555	46,927	30,124	28,996	27,418	5,277	5,751	5,093	4,900	5,814	6,009	5,721
Peru	1,323	5,517	6,176	7,864	7,418	5,000	3,118	9,213	12,107	3,874	3,836	5,801
Seaweed aquaculture^b^												
Argentina	3											
Brazil									320^c^	520^c^	730^c^	730^c^
Chile	33,471	65,538	71,648	40,079	20,273	15,493	33,586	23,668	21,687	88,193	12,179	14,469
México												
Peru	11	12										

*IFOP* Instituto de Fomento Pesquero (www.ifop.cl), *SAGARPA* Secretaría de Agricultura, Ganadería, Desarrollo Rural, Pesca y Alimentación (http://www.sagarpa.gob.mx), *DIREPRO* Dirección Regional de Produce (Regional Direction from Production Ministry)

^a^Harvest data from Chile (IFOP), Mexico (SAGARPA), Peru (DIREPRO)

^b^Aquaculture data from FAO (18/12/2013)

^c^FAO estimates

## State-of-the art on the management and regulation in Latin America seaweed industry

In Latin America, several species of red and brown seaweed are harvested as a raw material for phycocolloid extraction. Chile was one of the first agar producers in the world and supplies 10 % of the global biomass for alginates (Bixler and Porse 2011; Vásquez et al. 2012). In Chile, coastal communities have harvested primarily *Gracilaria* and *Lessonia* species since the 1980s, while in more recent years, *Macrocystis* species has been cultivated, first to feed abalone and most recently for bioenergy production (Buschmann et al. 2014). In Chile, several other benthic fisheries like sea urchins and molluscs depend on existence of seaweed beds. The exploitation of natural seaweed resources is therefore regulated with aims to protect the target species as well as the associated biodiversity. Several management strategies have been implemented successfully during the last 10 years, considering comanagement between fishermen unions and the state, using strategies with biological and ecological bases like morphological constraints, quotas by fishing areas, biological bans, rotation of harvesting, and also using experimental areas for harvesting and collection (Vásquez 2008; Vásquez et al. 2012). Lately, Vega et al. (2013) suggested an ecosystem approach and community indicators to manage wild brown seaweed stocks along the country.

**Fig. 1:**
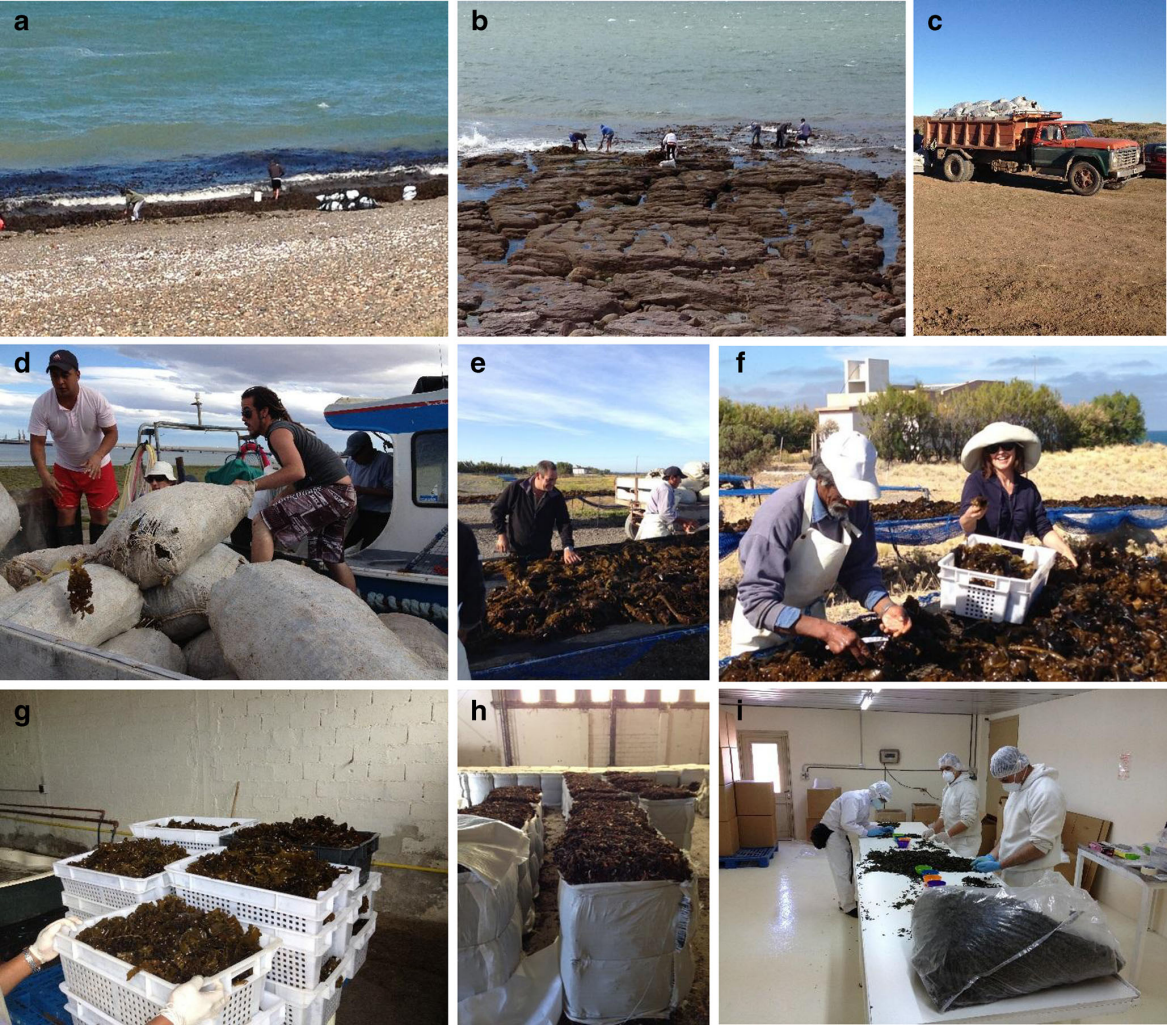
Argentina. Harvesting of seaweed in Patagonia. (**a**) Beach-casted seaweed is harvested. (**b**) Seabed of *U. pinnatifida* is harvested at low tide. (**c**) Transport in truck of the harvest biomass to the drying site. (**d**) Algae are harvested in nets. **e** Suspended net for drying the *U. pinnatifida*. (**f**) Selection of high-quality algae (processing of high-quality algae). (**g**) Preparation of high-quality *U. pinnatifida* for the Japanese market. (**h**) Storage of *Lessonia* sp. in bulk for the Chinese market. (**i**) Quality control on final product (photos: G Soriano)

In Argentina, different commercial species can be found such as *Undaria pinnatifida*, *Gracilaria gracilis*, *Gigartina skottsbergii*, *Lessonia vadosa*, *Macrocystis pyrifera*, *Porphyra columbina*, *Ulva lactuca*, and *Codium fragile*. In 1958, *G. gracilis* and *G. skottsbergii* were already harvested for the agar and carrageenan industries, respectively. Since 1980, *L. vadosa* and *M. pyrifera* are exported to the USA and China to supply the alginate industry, and since 1999, the uses of the Argentinian seaweeds have expended to new markets, such as for human consumption, nutraceuticals, and cosmetic including the fucoidan industries. All seaweed is harvested in Patagonia, mostly in the provinces of Chubut (Fig. 1a, b) and Santa Cruz. Local farmers directly sell the seaweeds to the processing companies or companies with concessions which directly employ their own workers for harvesting during the year and contracted divers in the summer (Fig. 1 c–i). Harvest is regulated since 1970 by local government through special licenses for 3, 10, or 30 years. The duration of the license is decided based on the investment of the companies. Today, there is only one company (Soriano SA) that has worked steadily through the years producing agar and carrageenan. The National Center of Patagonia (CENPAT) guarantees that the harvesting methods are performed in a sustainable way. Regulations for the management of brown seaweeds and marine concessions are particularly well developed, and the supply in brown seaweed to the alginate industry is well managed and organized (Zaixso et al. 2006; Soriano SA unpublished report).

**Fig. 2:**
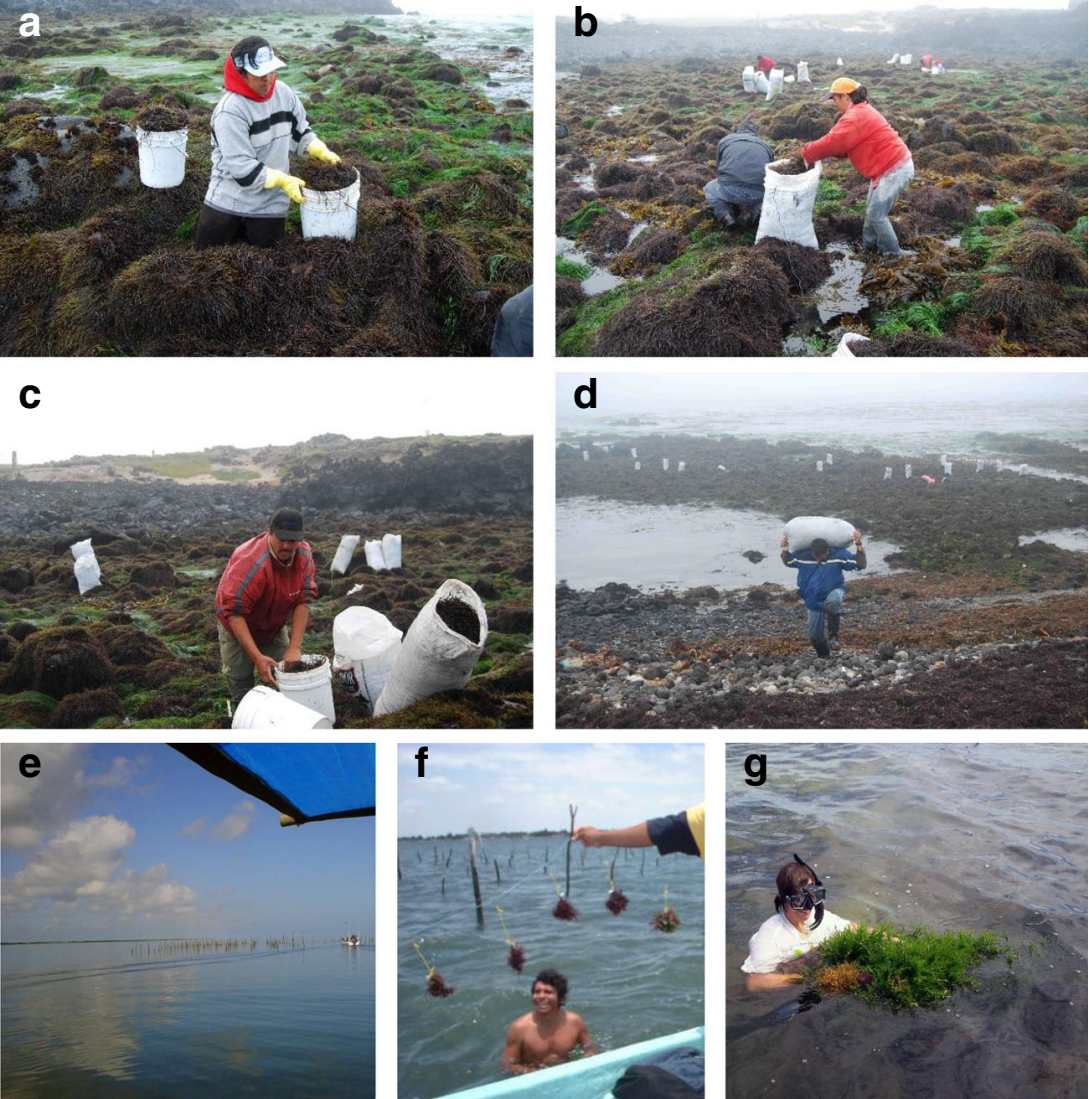
Mexico. (**a**–**d**) Harvesting of *Chondracanthus canaliculatus* in San Quintin, Baja California. *C. canaliculatus* is harvested by hand in low tide. In the harvest, men and women participate together. (Photos: J Zertuche). (**e**–**g**) *Kappaphycus alvarezii* aquaculture and harvesting in Dzilam de Bravo, Yucatan (photos: D Robledo)

In Mexico, the federal government manages all fisheries. However, under a law published in 2009, individual states can also manage sessile marine resources through an agreement to the federation. Presently, Mexico’s seaweed biomass is sold to the phycocolloid industry, abalone farming, and seaweed extracts for agriculture (DOF 2012; Zertuche-González et al. 2013). None of the four commercial seaweed species harvested in Mexico (*M. pyrifera*, *Gelidium robustum*, *Chondracanthus canaliculatus*, *Gracilariopsis lemaneiformis*) are endangered, thanks to the application of proper harvesting methods (Hernández-Garibay et al. 2006; Robledo 2006; DOF 2012). The highest reduction of the seaweed resource was observed along the Pacific Mexican coast during the years when El Niño occurred, but even then, the resources recovered successfully (Ladah et al. 1999; Casas-Valdez et al. 2003). In Mexico, seaweed harvesting may be classified into three broad categories of activity: by hand (at low tide), by diving, and by mechanized methods (specialized boats). Men and women participate both in harvesting and drying. Women and other family members are however mostly involved in the manual gathering, while men dominate the diving and mechanical harvesting, due to the hardship of the work. *C. canaliculatus* has been harvested for carrageenan, by hand, during low tide since 1966, and it is a sustainable harvest up to today (Fig. 2 a–d). The economic outcome from sustainable exploitation of natural resources does not yet necessarily assure a good livelihood to fishermen. Fishermen involved in seaweed harvesting by boat and diving (such as in the case of *G. robustum* and *M. pyrifera*) get better income than those harvesting by hand in low tide. *C. canaliculatus*, for instance, has been harvested for carrageenan, by hand, during low tide since 1966, and it is a sustainable harvest up to today (Fig. 2 a–d); however, harvesting and drying methods are artisanal and seasonal and therefore generate low income. Fishermen that harvest seaweeds from boats and diving gear complement their income by participating in other fisheries such as abalone or sea urchin. Although the seaweed fishery is economically or environmentally sustainable, the livelihood of the fishermen is not always attractive. It is therefore important to look at the fishery in the larger context, and the cultivation of seaweeds (as opposed to their harvesting) may actually offer a better alternative to coastal communities, as it can give them the opportunity to increase production and improve productivity and quality. In this regard, a socio-economic analysis of seaweed farming involving coastal communities was performed in the Gulf of Mexico coast using assumptions and data from the pilot cultivation of *Kappaphycus alvarezii* carried out in Dzilam, Yucatan Peninsula (Robledo et al. 2013). Based on this experience, various social and institutional factors in seaweed farming were discussed, which indicated a good potential for seaweed farming to become an integrated part of the local livelihood strategies for community development (Fig. 2e–g). One of the major conclusions of this study was that any integration of seaweed growing and industrialization in Mexico would require an interest from industry as well as local investors and government authorities in order to ensure successful implementation and development of the activity.

Peru is just starting to establish regulations for management or exploitation of the seaweed resources including some harvesting restrictions associated with the brown seaweed populations (Vásquez et al. 2012). Southern Peru has approximately 1,000 km of exploitable coastline, with several major, natural populations of brown seaweed species, such as *Lessonia nigrescens*, *Lessonia trabeculata*, *Macrocystis integrifolia*, and *M. pyrifera* (Acleto 2006). The Peruvian production was previously based on the harvesting of *Gracilaria* species. Until 2001, drift brown seaweeds were collected along the shoreline and directly sold to one processing company. McHugh (2002) reported that *L. nigrescens* and *L. trabeculata* were available from natural beds in the south of Peru, although the biomass was strongly affected by El Niño events (Taylor et al. 2008). In the years when the natural marine resources are not negatively affected by El Niño events, about 3,000 t of dry *L. nigrescens* and dry *L. trabeculata* is exported to Asia (Peruvian seaweed exports 2012, PSW SA unpublished report). *M. pyrifera* is also widely available, and prior to 2001, it was exported only in small quantities. In 2012, 95.5 % of the Peruvian seaweed exports was done by the four main Peruvian-based companies (one Chilean and three Chinese). Today, there are four companies purchasing seaweed from the local communities: two Peruvian companies, one Chilean, and one Chinese-owned company. The occurrence of Chinese-owned companies in the Peruvian market has triggered an intensive competition for obtaining the seaweed biomass from the local fishers. As captured fisheries (including seaweed) decline because of overharvesting, the prices of target species often increase dramatically. Likewise, the most commonly harvested fish in Peru, anchoveta (Engraulis ringens), has had a dynamic history of overharvest and fluctuating production (Diana 2009). As a result, beach prices of dried seaweeds increased from US$60 to US$400 t^−1^ between 2006 and 2008 (Table 2, PSW SA unpublished report), creating in the short term an uncontrolled intensification of the harvesting and resulting in a depletion of the natural resources that could have long-term consequences for the Peruvian fishers and Peruvian seaweed industry. Thus, while anchoveta indeed is the fundamental fish species in the Peruvian ecosystem, there are other fisheries to be considered for management (including seaweed). There are trade-offs in managing fisheries, and ideally, such trade-offs should be known when setting fisheries policies (Christensen et al. 2014). This area is well known for experiencing large changes in the abundance and species composition of its main fish resources. Three decades ago, the Peruvian fishery also experienced an economic and social crisis due to an absence of adequate management actions in relation to overexploitation of the anchovy resource (Schreiber 2013). Particular favorable environmental conditions, good recruitment coupled with careful management, and a surveillance scheme have apparently contributed to the fast recovery of the anchovy resource at the end of the 1990s, provided by the political regulatory organism for fisheries resource PRODUCE (Ministry of Production) with the information given by their scientific and technical entity Marine Institute of Peru (IMARPE).

**Table 2:** Prices in US dollars per ton of dried seaweed paid to the Peruvian fishermen excluding the tax (IGV 19 %; PSW SA unpublished data)

Species	Early 2007	Early 2008	At present
*Macrocystis pyrifera*	74	170	400
*Chondracanthus chamissoi*	440	–	840

**Fig. 3:**
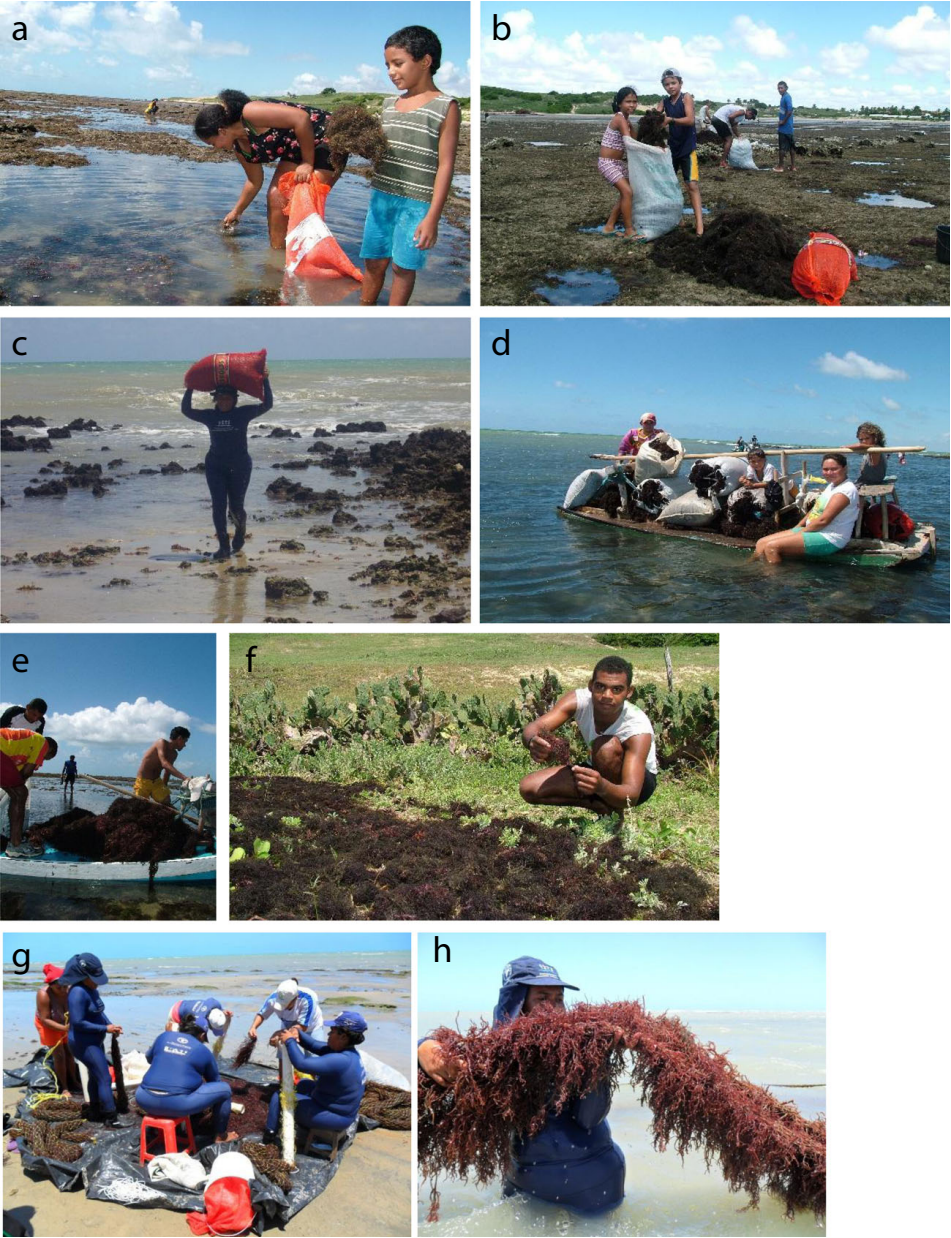
Brazil. Harvesting and aquaculture in the community of Rio do Fogo, Rio Grande do Norte, Brazil. (**a**–**b**) Women and their children harvesting the natural bed of seaweeds at low tide. (**c**–**d**) Gathering of the seaweed in bags. (**d**–**e**) Harvesting of the seaweed by boat. (**f**) Sun-drying of the seaweeds. (**g**) Preparation of the seaweed seedling net by the women of the community. (**h**) Culture line of *Gracilaria birdie* (photos: E Marinho-Soriano)

In the case of Brazil, historically, the seaweed industry is based on harvesting of seaweed from wild populations, a practice maintained until today. Industries based on *Kappaphycus* farming have also been established in the last 20 years but still need to address the issues relative to the acceptance of this new activity from the authorities (Hayashi et al. 2013). Despite the large number of economically commercial species, only few species of *Gracilaria* and *Hypnea* are exploited commercially in Brazil (Marinho-Soriano et al. 2006). Red seaweed exploitation began in the 1960s along the northeast coast and was undertaken without regulations. In 2006, the Brazilian Institute of Environment and Renewable Natural Resources (IBAMA) implemented a regulation on the harvest of natural resources along the coastline. However, the lack of taxation caused overexploitation of natural beds and most of activities that were then dependent on the harvesting of these species have nowadays stopped (Hayashi et al. 2013). Seaweeds are still a significant source of income and support the livelihoods of many people within coastal communities. Harvest is considered a secondary activity, undertaken without the participation of the family heads, who are generally fishermen (E Marinho-Soriano unpublished). On the other hand, for many women, harvesting is considered their main occupation, even if it is performed only part time, during low tide (Fig. 3a–c). The harvest is done on the shores or by diving from a small boat (“jangadas,” Fig. 3d, e), when the seaweed is further out. This activity is mostly carried out by women and their children equipped with a net bag (Fig. 3b, c). After the harvest, the biomass is brought back to the shore and then sun-dried for 2 to 3 days on land (Fig. 3f). Finally, the dried seaweed is sold to an intermediate collector and will be used for agar or carrageenan production (E Marinho-Soriano unpublished).

To improve the situation of poor coastal fishing communities of Brazil, in 2001, the Brazilian government started an FAO Technical Cooperation Project (TCP/BRA/0065) with the objective of assisting the establishment of a sustainable seaweed farming sector that could benefit poor coastal communities in the northeast states (Rio Grande do Norte, Paraiba e Ceará). The scope of the project was testing farming techniques in pilot communities and verifying the technical and financial feasibility of the packages, promoting the associative work among the producers, monitoring the social impact of the introduction of these new techniques, and facilitating the establishment of an institutional framework for this new production sector (Freddi and Aguilar-Manjarrez 2003). Moreover, the project showed that community members easily learned techniques for seaweed cultivation, and red seaweed farming (*Gracilaria birdiae*) became a significant source of income, particularly for the poorer segments of the coastal fishing population (Fig. 3g, h). A fivefold increase in the prices of dry seaweed collected from natural beds or from the beaches was experienced by the seaweed-farming industries (Freddi and Aguilar-Manjarrez 2003). Nowadays, the kilogram of dried *Gracilaria* is sold for US$3.50; seaweeds can therefore bring to the coastal communities an additional income as described in the last data published by the Brazilian Institute of Geography and Statistics (IBGE 2014).

In Brazil, the size of the coastal zone and the large ecosystems and diversity produce a false idea of an inexhaustible exploitation potential, causing adoption of development policies that do not take into account the sustainable use of the resources (MMA/REVIZEE 2006). As a result, over 80 % of the resource is currently overexploited (Miloslavich et al. 2011). The importance of the fishing industry in Brazil is, however, incontestable. Marine fisheries contribute with 63 % of the national fish production and involve approximately 800,000 professionals creating 3.5 million jobs directly or indirectly on the sector (ACEB 2014). According to the IBGE (2014), approximately one quarter of the Brazilian population lives in the coastal area, accounting 50.7 million residents. The process of urbanization in which population and economic activities are concentrated particularly along the coastline has been one of the main processes responsible for the modification of habitat and communities, as well as increasing pressures on water resources (Marques et al. 2004). Only a small portion of the enormous Brazilian coastline is under some form of protection or management, and large areas are subject to anthropogenic pressures, encroachments, and overuse (Amaral and Jablonski 2005).

While Mexico, Chile, and Argentina have developed coastal management plans and appropriate regulations for harvesting of their natural resources, Peru and Brazil do not have well-established regulations or sustainable practices in regard to the exploitation of their natural seaweed resources to avoid depletion of their natural beds (Santelices and Doty 1989; Vásquez et al. 2012). Due to the significance of the seaweed forest for coastal ecosystems and its place in the maritime food chain, a widespread reduction in South American seaweed resources may have an impact on the overall productivity and stability of the coastal communities, with only moderate capacity to adopt to societal and environmental changes. In Peru, the Humboldt Current Large Marine Ecosystem (HCLME) Project therefore attempts to develop terms of reference and comprehensive plan of action (PIA), for the repopulation of natural seaweed beds along the Peruvian coast. In the Bay of Paracas ecosystem (Ica Region, Peru), through laboratory cultivation, transplanting, and harvesting, the PIA enables the recovery of the kelp populations, which were negatively affected by indiscriminate harvesting. The PIA also allows for the sustainability of the economic activity for the community of artisanal fishermen in the area in order to start a sustainable production that includes the transfer of appropriate technologies to them. The understanding of the synergy between ecology, economy, and sociology involved in the exploitation of selected seaweeds provides a sustainable platform for seaweed exploitation in Latin America (Clausen and York 2008).

## European and American experiences: what are the “lessons to learn” on regulations and management for a long-term sustainable seaweed industry in Latin America?

In Europe, the Norwegian seaweed industry is producing over 60 % of the biomass and is almost completely reliant on natural beds of *Ascophyllum nodosum* and *Laminaria hyperborea. L. hyperborea* accounts for about 90 % of the national harvest (Meland and Rebours 2012a), and the alginate industry is by far the most important sector. The remaining biomass is used directly for food, fodder, cosmetics, and the health sectors. Regulations for harvesting seaweed (Meland and Rebours 2012b) are applied to seabed algae such as *L. hyperborea*, and private owner rights only regulate the harvest of foreshore algae such as *A. nodosum*. However, environmental protection laws and other regulations can restrict areas for harvesting. Even a strong interest for seaweed aquaculture, particularly as part of integrated multitrophic aquaculture (Leonczek 2013), the development of any new industrial sector in Norway follows the principle of precaution and is for now highly restricted and controlled by the existing regulation (Alexander et al. 2014) as regulations are under development (Meland and Rebours 2012b).

Portugal seaweed activities are now reduced to the seasonal and low-volume harvest of agarophyte and carrageenophyte species, whereas in the 1980s it was one of the world’s largest agar producer (Sousa-Pinto and Araújo 2006). Despite the existence of several economical valuable species, regulations only exist for residual (once important) activities such as Gelidium harvesting and beach cast seaweed activities. A revision of the existing regulation that allows the exploitation of new species is required not only in order to establish sustainable harvesting activities but also to prevent the uncontrolled harvest of economically important sensitive species (Araújo et al. 2009). On the other hand, the country’s recent focus on aquaculture development opens the door for seaweed cultivation in monoculture or integrated multitrophic aquaculture (IMTA) systems (Abreu et al. 2011a).

Commercial exploitation of seaweeds in eastern Canada began in the early 1940s with the harvest of the carrageenophyte *Chondrus crispus*. From 1965 to 1980, Canada was the world’s main supplier of raw material for the carrageenan industry, with a peak landing over 50,000 wet t in 1974. By the 1990s, the landings sharply declined to less than 10,000 wet t due to a combination of international competition and biological fluctuations in the abundance of the resource (Chopin and Ugarte 2006). Today, *A. nodosum* is the main economic seaweed resource in Canada, with over 40,000 t landed in 2010 (Ugarte and Sharp 2011). This seaweed is being used for among other applications as a biostimulant extract for crops and animal feed supplements, incorporating more than US$40 million to the local economy. *A. nodosum* has been managed since 1995 under an ecosystem approach, following strict regulations established in the Ocean Act (Ugarte and Sharp 2001). To solve problems mainly of fluctuating supply and avoid the risks of overexploitation, the aquaculture of *C. crispus* was developed in the 1970s. *C. crispus* production is the main commercial Canadian seaweed aquaculture, with the culture of *Porphyra* species and *Saccharina latissima* as organic extractive components of integrated multitrophic aquaculture system (Levine 2004; Chopin and Ugarte 2006; Chopin 2008).

Like other European and American countries, the Brazilian and Peruvian dependency on traditional coastal fisheries and eventual growth in aquaculture is followed by an urgent need for robust institutions for planning and managing the coastal zone with focus on developing an integrated and sustainable management plan for the seaweed resources (Prates 2007; Castello 2010; Johnsen et al. 2014). The steep growth in the use of marine biological resources represents a fundamental change in the way humans derive benefits from the oceans. Marine aquaculture production is growing at 7 % per year and the sector of domestication of marine species is growing at about 3 % per year, while the number of natural marine products of commercial interest is growing at a rate of 4 % per year (Duarte et al. 2007). Large-scale integrated conservation and management plans are thus urgently required in order to address the sustainable development of various economic and societal sectors. In addition to developing and implementing management plans for sustainable use, these countries also face huge tasks connected to mapping, investigating, and monitoring their marine biological diversity. Information and “lessons learned” may be provided by the scientific communities and management plans in Latin American countries such as Chile or Mexico (Vásquez et al. 2006; 2012; Ávila et al. 2013; Vega et al. 2013) and European directives such as those from Norway (Meland and Rebours 2012a) or the ecosystem-based management regulated by the Ocean Act in Canada (Ugarte and Sharp 2001). National regulations should be established on best practices for seaweed harvesting, management, and cultivation, and the knowledge should be passed on to the coastal communities, which could then make seaweed a sustainable opportunity for income.

Alternatively, the seaweed biomass could be obtained through cultivation (McHugh 2002). The seaweed aquaculture industry still requires technological and management improvements, institutional changes, and appropriate environmental and social frameworks (Valenti 2008; Oliveira 2009; Abreu et al. 2011b; Marroni and Asmus 2013). As seaweed aquaculture is at its infancy both in Europe and America, its development will have to overcome numerous challenges by introducing innovations at different levels, i.e., technology, biology, and policy. However, cultivation techniques are standardized and economically sustainable especially in Asia (Miura 1980) and cultivated macroalgae now supply 96 % of the global demand (FAO 2014). The aquaculture of macroalgae is also strongly recommended by the EU regulation (EC No 710/2009) for the organic production of animals in aquaculture systems. The development of regulations for supporting the aquaculture of seaweed is a necessary action in recent years, and these regulations should be guided by best practices. The combined effect of rapidly increasing domestication and production with increasing demand for seaweed products is likely to promote innovation in seaweed biotechnology, and these efforts must be accompanied by substantial scientific effort (Mazarrasa et al. 2013). In addition, revision of the Common Agricultural Policy (CAP) intends to include management with High Nature Value in organic certification. Nordic countries such as Denmark, Sweden, and Norway are also aiming to increase organic products to 15 % of the home market. Several food and food supplement products directly prepared from algae are already certified in Europe and in Norway as organic food and given opportunities for producers to access to a niche market (Friis Pedersen et al. 2013).

## Steps towards wealth and sustainable livelihoods for Latin America coastal communities

The seascapes are increasingly managed for multiple functions and services in addition to provision of food, and this requires the integration of ecological and socio-economic research, policy innovation, and public education. The multiuse dilemma has driven many researchers, experts, and policy makers to try and address issues related to the sustainability of coastal development from disciplinary/sectorial perspectives addressing the interactions and functioning within the wider ecosystem, social, economic, and political contexts (Buchholz et al. 2012). A review by Harley et al. (2012) addressed the significant gaps in understanding, which hamper ability to predict the outcomes of global change in seaweed-dominated systems. In particular, it addressed the lack of general or even basic understanding of (a) the importance of rates, timing, magnitude, and duration of environmental change; (b) nonadditive effects of multiple stressors; (c) population-level implications of variable environmental impacts among life history stages; (d) the scope for population- or species-level adaptation to environmental change; and (e) ecological responses at the level of communities and ecosystems, including tipping points and sudden phase shifts. In this regard, biological responses (i.e., ecophysiological) to key environmental drivers or combinations of drivers can be incorporated into demographic models to better describe and predict changes in population growth or decline. The expansion of seaweed cultivation particularly in tropical regions contributes significantly to carbon sequestration since the rapid turnover in seaweed culture, approximately 3 months per crop (in the tropics) with yields of over 2,500 wet t ha^−1^ (De Silva and Soto 2009; Vásquez et al. 2013). Nevertheless, some authors have pointed out that a significant proportion (estimates range up to 60 %) of the carbon they fix photosynthetically is released into the water, and a proportion of this released dissolved organic carbon (DOC) is highly labile, entering in the bacterial loop and rapidly remineralizing back to CO_2_ (Hughes et al. 2012). Environmental impacts of seaweed farming in the tropics have been reviewed by Zemke-White and Smith (2006). Some authors have also pointed out other environmental impacts of algal farming both positive (i.e., increase on fish assemblages; Bergman et al. 2001) and/or negative (i.e., effect on the meiobenthos; Olafsson et al. 1995). All of these impacts should be considered when the environmental effects of seaweed aquaculture are taken into account. The concept of ecosystem-based management approaches based on an integrated approach of entire ecosystem including humans should also be considered to develop the coastal spatial planning and the best practices guidelines for the exploitation of seaweed (both harvesting and aquaculture) in order to avoid spatial and temporal mismatches of the governance (Crowder and Norse 2008).

The benefits for the well-being of coastal communities as increase of direct permanent employment in previously disadvantaged coastal communities, where unemployment is not only an economic issue but also a socio-political concern, have been exemplified in an IMTA farm of abalone and seaweed (Nobre et al. 2010). Sustainable management of coastal resources creates new economic activities based on the exploitation of a raw material and could participate in the local socio-economic development in coastal areas and communities in Latin America. Developing long-term management plans will also produce fundamental long-term results of interest for the international research community. Socio-economic benefits derived from seaweeds have been already exemplified in the Philippines where approximately 116,000 families consisting of one million individuals were farming more than 58,000 ha of seaweed, making seaweed farming the largest and most productive form of livelihood among the coastal population. In Zanzibar (Tanzania), more than 90 % of seaweed farmers are women. Like in Latin America, changing the life in the villages by women gaining independent economic power will contribute in reducing childhood malnutrition (as an indicator that the health of their mothers has improved), increasing the numbers of children attending schools regularly, and reversing the trend of rural depopulation by self-employment of the village youths (Msuya 2006; Msuya et al. 2007).

Innovation should be promoted when trying to integrate seaweed harvesting or aquaculture as part of the wealth for coastal communities. In this regard, Castellacci (2010) pointed out that the technology dynamics of a country depend on three main factors: its innovative intensity, its human capital, and its technological infrastructures. Nowadays, Latin American countries show a much lower innovative strength than Norway or Canada, where the innovation gap has been quantified with a ratio of 16:1 for patents and 10:1 for scientific articles (Castellacci 2010). In order to get closer and eventually jump to the innovation development stage, developing economies such as in some Latin American countries should implement an appropriate combination of policies that takes into account the need to simultaneously develop R & D activities, traditional infrastructures, information and communication technologies, and advanced human skills. Human capital education explains differences in economic performance across countries; education is therefore a necessity to promote social inclusion and cohesion as well as employment. By focusing on marine resources involving low-cost technology requirements, such as the production of seaweed, developing countries are provided an opportunity to access an emerging market, propelled by a diversification of the demand for seaweed products from traditional uses to bioenergy, cosmetics, and biomedicine applications.

## Conclusions

The European, Canadian, and Latin American seaweed industries rely on the harvesting of their natural resources—activity limited by the biomass availability and the potential for the harvested species to recover. Different scenarios associated to seaweed resource exploitation can be observed, and several countries are in need of long-term management plans for the sustainable exploitation of their natural seaweed (Hersoug and Revold 2012). As this activity increases, there is also an urgent need to develop and implement ecosystem-based management models and integrated coastal zone planning. Policy makers must develop regulations and directives that enable a sustainable exploitation of the natural resource, not only to preserve marine and coastal ecosystems but also to ensure social stability and economic income of local communities.

Both European and Latin American countries need to address capacity building and adaptive governance towards seaweed resources. Norway and Canada have well-established management regimes for the sustainable exploitation of their seaweed resources based on the development and application of a sound knowledge and cross-sectorial management and spatial plans. Portugal, on the contrary, needs to review the national and regional management plans concerning the exploitation of the seaweed resources. In Latin America, it is important to stress that Chile has succeeded in establishing a sustainable harvesting in the south of the country. The further expansion of the seaweed industry in this region thus depends on reliable access to raw material, the development of value-added products, and the transfer of expertise between developed and less developed regions. An alternative solution to produce seaweed biomass for this growing sector is offered by aquaculture, which will also require guidelines for evolving into a responsible and well-managed farming.

Finally, trained human resources are required in order to provide education to coastal communities, based on best practices for harvesting and cultivation, in order to establish profitable businesses which could provide socio-economic development leading to better living conditions to the coastal rural communities. The current weak participation of developing countries in the seaweed market could then be reversed by strengthening cooperation to transfer technological knowledge and investment to developing nations active in seaweed management and aquaculture, thereby promoting sustainable development based on their own natural resources, as encouraged by the Convention on Biological Diversity (http://www.cbd.int/doc/legal/cbd-en.pdf). In this context, South American, European, and North American experts should therefore collaborate to develop guidelines to plan an integrated coastal management for Latin American seaweed resources. It is also of great importance for the legitimacy of these guidelines that these originate from transnational and cross-sectorial cooperation, including political, cultural, commercial and industrial actors, NGOs, and research communities. As a first action of knowledge transfer, research and technology development (RTD) and small and medium enterprises (SME) representatives from Norway, Argentina, Brazil, Canada, Chile, Mexico, Peru, and Portugal have established a common working framework in order to support the development of the Latin American seaweed sector. These industry/academia networks encourage cooperation among the seaweed stakeholders; across the project areas; in all aspects related to seaweed production, research, ecosystem services, and management of artisanal and small-scale aquaculture; and traditional and alternative market and economics.
